# The Incidence Rate, Risk Factors and Clinical Outcome of Acute Kidney Injury in Critical Patients

**Published:** 2018-11

**Authors:** Susan MOHAMMADI KEBAR, Saeed HOSSEINI NIA, Nasrollah MALEKI, Afshan SHARGHI, Arash SHESHGELANI

**Affiliations:** 1. Dept. of Nephrology, School of Medicine, Ardabil University of Medical Sciences, Ardabil, Iran; 2. Dept. of Internal Medicine, School of Medicine, Ardabil University of Medical Sciences, Ardabil, Iran; 3. Dept. of Hematology, Oncology and Bone Marrow Transplantation, Shariati Hospital, Tehran University of Medical Sciences, Tehran, Iran; 4. Dept. of Community Medicine, School of Medicine, Ardabil University of Medical Sciences, Ardabil, Iran

**Keywords:** Acute kidney injury, Intensive care units, Incidence, Mortality, Outcome

## Abstract

**Background:**

Acute kidney injury (AKI) is the most common cause of organ dysfunction in intensive care unit (ICU) patients. There is no consensus definition of AKI in ICU patients. Therefore, we aimed to evaluate the incidence rate, risk factors and clinical outcome of AKI using the RIFLE (Risk, Injury, Failure, Loss of kidney function, and End-stage kidney disease) classification in ICU patients.

**Methods:**

We performed a retrospective cohort study, on 900 patients admitted to the ICU during a one year period at Imam Khomeini Hospital in Ardebil, Iran from 2014 to 2015. AKI was defined by the consensus RIFLE criteria.

**Results:**

The overall incidence rate of AKI was 37%. The patients with AKI were also classified according to RIFLE as follows: Risk (8.2%), Injury (13.4%), Failure (13.2%), Loss of kidney function (1.3%), and End-stage kidney disease (0.8%). The mortality rate was 58.3% for AKI patients, and 13.4% for non-AKI patients (*P*<0.001). Patients in RIFLE-R (Risk) had a mortality rate of 37.8% compared with 48.8% for those in RIFLE-I (Injury) and 76.5% for RIFLE-F (Failure) patients (*P*<0.0001). Significant risk factors to the development of AKI were included: age more than 60 yr, increased length of hospital stay, systolic blood pressure less than 100 mm Hg, requirement of mechanical ventilation, relevant comorbidities, anemia, thrombocytopenia, increased serum bilirubin and liver enzymes, and serum sodium abnormalities.

**Conclusion:**

The RIFLE classification is a useful and suitable clinical tool to evaluate the incidence and mortality rate of AKI. In ICU patients, AKI is associated with increased mortality rate.

## Introduction

Acute kidney injury (AKI) is a major public health problem that affects millions of patients worldwide and leads to decreased survival and increased mortality risk, progression to chronic kidney disease, and functional status in daily living (1). AKI is the most common cause of organ dysfunction in intensive care unit (ICU) patients. The incidence of AKI is increasing. Among the studies that adopted a Kidney Disease Improving Global Outcomes (KDIGO) - equivalent AKI definition, AKI affects 20.9% of patients admitted to the hospital settings and 31.7% of patients in critical care settings (2).

There is no consensus definition of AKI in ICU patients. In response to the lack of a standard definition for AKI, the Acute Dialysis Quality Initiative (ADQI) group developed a consensus definition and classification of AKI: the RIFLE (Risk, Injury, Failure, Loss of kidney function, and End-stage kidney disease) classification system (3). The RIFLE classification is based on serum creatinine and urine output determinants and considers three severity classes of AKI (Risk, Injury and Failure), and two outcome classes (loss of kidney function and end-stage kidney disease). More than two-thirds of all ICU patients will develop AKI defined by the RIFLE classification. In addition, AKI in ICU patients is associated with a prolonged hospital stay, progression to chronic kidney disease, and a higher risk of in-hospital mortality. Increasing RIFLE class is associated with increasing risk of in-hospital death (4). The most important limitation of the RIFLE classification is that baseline serum creatinine is necessary for the definition and classification of AKI. Nevertheless, a more recent classification for AKI has been proposed by the Acute Kidney Injury Network (AKIN). It reduces the need for baseline creatinine but does require at least two creatinine values within 48 h (5).

The RIFLE classification is still commonly used as the monitoring of the progression of AKI severity in critically ill patients admitted to ICU and has been validated in many research and clinical audit purposes. In fact, the AKIN classification compared with the RIFLE classification did not exhibit a better prognostic acuity in terms of in-hospital mortality, although it enabled the identification of more AKI patients (6).

There have been many studies about the epidemiology and risk factors of AKI in critically ill patients in the different regions of the world and most data available are from developed countries (7, 8). The incidence and risk factors of AKI in developing countries may be different from those of developed countries. Therefore, extrapolation of results from other countries will not give a valid estimate of incidence and risk factors of AKI in Iran. Information on overall incidence, risk factors and outcome of AKI could help in developing strategies for prevention and treatment of AKI.

To our knowledge, little data on the incidence and risk factors for development of AKI in critically ill patients are available in Iran, and therefore the objective of this prospective study was to evaluate the incidence rate, risk factors and clinical outcome of AKI using the RIFLE classification in patients admitted in the ICU.

## Materials and Methods

### Study Setting

The study was approved by the Ethics Committee of the Ardabil University of Medical Sciences (Institutional approval Code: IR.ARUMS.REC.1394.12). Informed consent was obtained from all individual participants included in the study.

We prospectively performed a study of all ICU patients over a period of 12 months from Dec 2014 to Dec 2015 at Imam Khomeini Hospital, Ardabil University of Medical Science, Ardabil, Iran. Imam Khomeini Hospital is an educational and government institution in southern Iran serving a population of 1,248,488. With 549 nominal bed counts, it provides health care services for all of the citizens of north-west of Iran.

### Study Population

All patients who admitted to the ICU with a length of stay greater than 48 h were included in the study. During the study period, 900 patients were admitted to the ICU and the records of all patients were reviewed. We excluded patients who remained in the ICU for less than 48 h, patients with a history of chronic kidney disease, patients who admitted to the cardiac ICU, and those readmitted to the ICU.

### Data collection

A data collection sheet was prepared to summarize the information obtained from each patient record, including the demographic information (age, gender, etc.), clinical presentation, cause of admission, temperature, smoking, duration of hospitalization, patient's condition, the use of invasive procedures, respiratory aids, prescription antibiotics, pre-existing comorbidities, the possible causes of AKI, physiological variables and laboratory results. In patients discharged alive from the hospital, patients were followed up 3 and 6 months after hospital discharge by telephonic interview. Information on patient survival or date of death was obtained from the hospital registry office. The primary outcome of the study was 3-month and 6-month mortality.

### Definitions

Patients who met any of the criteria of the RIFLE classification were defined as AKI patients. AKI was considered if there was an increase in serum creatinine to more than 1.5-fold of baseline value, or a decrease in GFR to more than 25% from baseline value, or a urinary output lower than 0.5 mL/kg/h for 6 h. According to the status of serum creatinine and urine output, AKI was classified into three categories based on severity (Risk, Injury and Failure) and two categories based on clinical outcome (Loss of kidney function and End-stage kidney disease) (3) (Table 1).

**Table 1:** The RIFLE classification for acute kidney injury

***Classification***	***Serum Creatinine Criteria***	***Urine Output Criteria***
Risk	Increase in serum creatinine ≥ 1.5 × baseline or decrease in GFR ≥ 25%	< 0.5 mL/kg per hour × 6 h
Injury	Increase in serum creatinine ≥ 2.0 × baseline or decrease in GFR ≥ 50%	< 0.5 mL/kg per hour × 12 h
Failure	Increase in serum creatinine ≥ 3.0 × baseline or serum creatinine ≥ 4.0 mg/dL (354 μmol/L) or decrease in GFR ≥ 75%	< 0.3 mL/kg per hour × 24 h or anuria × 12 h
Loss of kidney function	Complete loss of kidney function > 4 wk	-
End-stage kidney disease	End stage renal disease (> 3 mo)	-

### Statistical analysis

The statistical analysis of the data was done using the SPSS software for Windows, version 21 (SPSS Inc., Chicago, IL, United States). All tests were two-sided and results were considered statistically significant if *P*-value was less than 0.05. Quantitative variables were expressed as means ± standard deviation (SD), and qualitative variables as percentage. The comparison of the continuous variables was accomplished with Student's t or Mann-Whitney U tests, and, for the comparison of the categorical variables, the Pearson chi-squared test (χ^2^ test) and Fisher exact test were used.

The association between development of AKI and possible risk factors was initially assessed with bivariate analyses (Pearson correlation). The potential risk factors were age, sex, smoking status, length of hospital stay, blood pressure, requirement of mechanical ventilation, relevant comorbidities, serum bilirubin, liver enzymes, complete blood count (CBC), prescribed drugs, and serum concentration of the sodium and potassium. Variables were included in the multivariate analysis if they had a P value of equal to or less than 0.20 in the bivariate analysis. The adjusted odds ratio (OR) with 95% confidence interval (CI) was calculated by multivariate logistic regression analysis. A *P*-value less than 0.05 was considered statistically significant.

## Results

Overall, 504 (56%) were men, and 264 (29.3%) of the patients had at least one comorbidity. Relevant comorbidities included hypertension (n=47, 17.8%), pulmonary disease (n=45, 17.1%), cardiovascular disease (n=40, 15.1%), AKI (n=37, 14.1%), septic shock (n=28, 10.6%), cerebrovascular accident (n=20, 7.5%), ventilator-associated events (n=25, 9.4%), chronic kidney disease (n=14, 5.3%), and diabetic ketoacidosis (n=8, 3.1%). The mean length of hospital stay was 8 ± 1.2 d.

### Incidence of AKI

The patients were divided into two groups based on the RIFLE classification: AKI group consisted 333 (37%; 95% CI, 33.9 to 40.2) patients with AKI, and non-AKI group consisted 567 (63%; 95% CI, 59.8 to 66.1) patients without AKI. The AKI group was also classified according to RIFLE as follows: Risk (n=74, 8.2%), Injury (n=121, 13.4%), Failure (n=119, 13.2%), Loss of kidney function (n=12, 1.3%), and End-stage kidney disease (n=7, 0.8%).

### The relationship between age and sex with incidence of AKI

Overall, the average age of patients was 54.7±8.2 yr (ranging from 25–93). Most of the patients (n=396, 44%) were in the age range 60–80 yr. Among these, 396 (44%) were women and 504 (56%) were men. The average age of the AKI group was 63±5.4 yr, and the average age of the non-AKI group was 45.1±4.9 yr. There was statistically significant difference between age and the incidence of AKI (*P-*value<0.05). Indeed, older age was a risk factor for the development of AKI.

In the patients with AKI, 200 (60.1%) were male and 133 (39.9%) were female. In the non-AKI group, 304 (53.6%) were male and 263 (46.4%) were female. However, no significant relationship was found between sex and the incidence of AKI (*P*=0.24) (Table 2).

**Table 2:** Characteristics of all patients admitted to the ICU (AKI and Non-AKI patients)

***Characteristics***	***Total patients (n=900)***	***Non-AKI patients (n=567)***	***AKI patients (n=333)***	***P-value***
Mean age (yr)	54.7 ± 8.2	45.1 ± 4.9	63 ± 5.4	<0.05
Gender	Male: 504 (56%)	Male: 304 (53.6%)	Male: 200 (60.1%)	
	Female: 396 (44%)	Female: 263 (46.4%)	Female: 133 (39.9%)	0.24
Smoker	171 (19)	94 (16.8)	77 (23.1)	0.18
Mortality rate; n (%)	270 (30)	76 (13.4)	194 (58.3)	<0.001

### The relationship between smoking and incidence of AKI

Of all patients, 171 (19%) were active smokers and 729 (81%) were non-smokers. In patients with AKI, 77 (23.1%) were active smokers. In the non-AKI group, 94 (16.8%) were active smokers. However, no significant relationship was found between smoking and incidence of AKI (*P*=0.18).

### The relationship between laboratory abnormalities and incidence of AKI

Among the 333 patients with AKI, 62.7% had anemia, 60% had hyperbilirubinemia, 62.2% had increased liver enzymes, 41.8% had sodium disturbances, 38.9% had thrombocytopenia, and 42.2% had potassium disturbances. There was statistically significant difference between anemia, hyperbilirubinemia, increased liver enzymes, sodium disturbances, and thrombocytopenia with the incidence of AKI (*P*<0.05). However, no significant relationship was found between potassium disturbances and the incidence of AKI (*P*=0.48).

### Outcome and mortality rate

The overall mortality among patients studied was 30% (270/900). The mortality rate was 58.3% (194/333) for AKI patients and 13.4% (76/567) for non-AKI patients. There was statistically significant difference between mortality and the incidence of AKI (*P*<0.001).

Based on the RIFLE classification, the mortality rate was 37.8% (28/74) for patients with Risk, 48.8% (59/121) for patients with Injury, 76.5% (91/119) for patients with Failure, 83.3% (10/12) for patients with Loss of kidney function, and 85.7% (6/7) for patients with End-stage kidney disease. A significant relationship was found between the different classes of RIFLE and mortality rate (*P*<0.0001) (Table 3).

**Table 3:** The outcome of patients by RIFLE classification

***Classification***		***Number of patients (%)***	***Mortality rate (%)***
Patients without AKI	Risk	567 (63)	13.4
		74 (8.2)	37.8
Patients with AKI	Injury	121 (13.4)	48.8
	Failure	119 (13.2)	76.5
	Loss of kidney function	12 (1.3)	83.3
	End-stage kidney disease	7 (0.8)	85.7

**Fig. 1:**
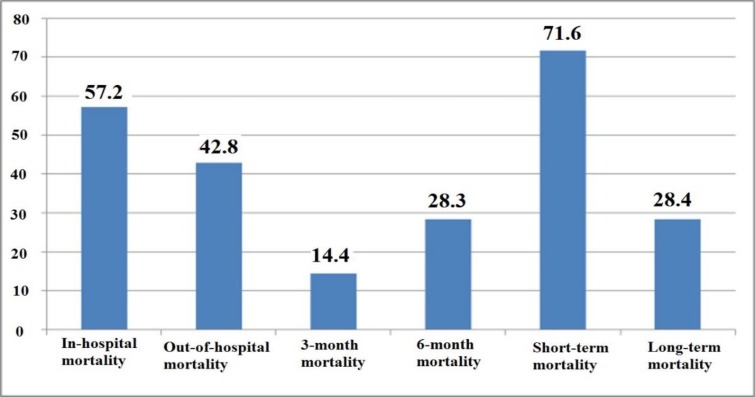
Overall 6-month mortality rate (both in-hospital and out of hospital) for Acute Kidney Injury

Out of 194 non-survivors in the AKI group, 111 (87.2%) died in the hospital, and 83 (42.8%) died outside the hospital. In-hospital and out-of-hospital mortality rate was 71.6% and 28.4%, respectively (Fig. 1). While a significant relationship was not found between the different classes of RIFLE and short-term mortality (*P*=0.12), it was a significant relationship between the REFLE-F class and short-term mortality (*P*<0.001), so that in patients with REFLE-F class, 60-day and 6-month mortality was 53.8% and 85.7%, respectively, which show a high level of short-term mortality in patients with REFLE-F class.

### Risk factors to the development of AKI

In the logistic regression analysis, significant risk factors for the development of AKI were included: age more than 60 yr (*P*<0.05), length of hospital stay over 6 months (*P*<0.0001), systolic blood pressure less than 100 mmHg (*P*<0.0001), requirement of mechanical ventilation (*P*<0.0001), relevant comorbidities (*P*=0.006), hemoglobin less than 10 g per decilitre (*P*<0.05), platelet count less than 100000 per microliter (*P*<0.05), increased serum bilirubin and liver enzymes (*P*<0.05), and serum sodium abnormalities (*P*<0.05). However, gender (*P*=0.24), serum potassium abnormalities (*P*=0.48), and prescribed drugs (*P*=0.18) were not statistically significantly associated with the incidence of AKI (Table 4).

**Table 4:** Multivariable logistic regression analysis of risk factors of acute kidney injury

***Predictor Variable***	Odds Ratio (95% CI)^*^	***P-value***
Age more than 60 yr	1.77 (1.17–2.68)	<0.05
Gender	0.83 (0.39–1.75)	0.244
Length of hospital stay over 6 months	1.93 (1.31–2.84)	<0.0001
Systolic blood pressure less than 100 mm Hg	2.71 (1.50–4.89)	<0.0001
Requirement of mechanical ventilation	1.20 (0.75–1.91)	<0.0001
Relevant comorbidities	1.27 (0.86–1.86)	0.006
Anemia	1.70 (0.97–2.90)	<0.05
Thrombocytopenia	1.31 (0.54–3.18)	<0.05
Increased serum bilirubin	1.06 (0.60–1.80)	<0.05
Increased liver enzymes	0.86 (0.43–1.70)	<0.05
Serum sodium abnormalities	1.12 (0.37–3.39)	<0.05
Serum potassium abnormalities	0.88 (0.61–1.26)	0.481
Prescribed drugs	0.79 (0.59–1.06)	0.183

* The reference for the odds ratio is the absence of the corresponding risk factor. Variables were entered into the multivariable logistic regression model (and, therefore, odds ratios were calculated) when the alpha level of the risk factor was less than 0.20 in bivariate analysis

## Discussion

This prospective cohort study offers valuable information for patients admitted to the ICU. First, our results confirm the high incidence of AKI in the studied population. Hence, the incidence of AKI based on the RIFLE classification in ICU patients was 37%. Second, although both AKI and non-AKI patients were associated with a higher mortality in our study, the mortality rate was significantly higher in AKI patients (58.3%), compared to non-AKI patients (13.4%). Last, our study provides significant risk factors for the development of AKI.

Multiple studies have evaluated the incidence, risk factors, and outcomes of AKI. For example, a meta-analysis of 154 cohort studies, involving 3,585,911 patients, conducted to estimate the world incidence of AKI according to the KDIGO staging system. The authors found the incidence rate of AKI to be 21.6% in adults and 33.7% in children, and a mortality rate of 23.9% in adults and 13.8% in children. This meta-analysis showed reduced mortality rate of AKI-related during the span of eight yr. In addition, the mortality was inversely related to income of countries and the percentage of gross domestic product spent on total health (2).

The incidence of AKI in our population was higher compared with those reported in Italy (10.8%) (9), Australia (11.5%) (10), Finland (19.3%) (11), Portugal (50%) (12), and Korea (54%) (13), but lower compared with those reported in Japan (73.1%) (14) and USA (67.2%) (15). The high mortality in the studied population can happen for several reasons. First, we may offer poor quality care to our ICU patients. Second, the RIFLE classification may need to be modified for use in our patients. Third, AKI patients in our study have a worse prognosis on ICU admission. Fourth, the increased sophistication and managing by more doctor groups may have led to this unfavorable outcome via high and unnecessary procedures and instrumentations and overlaps between the managing groups originating from a managing discrepancy. Finally, the better-equipped centers in other parts of the world may have been more aggressive in initiating dialysis.

The RIFLE classification is useful for identifying patients at greatest risk of adverse short-term outcomes. For example, in the United Kingdom, in a geographical population base of 523,390, the incidence of AKI and acute-on-chronic renal failure to be 1811 and 336 cases per million population, respectively (16). In this study, the RIFLE classification was useful for predicting a full recovery of renal function (*P*<0.001), renal replacement therapy requirement (*P*<0.001), length of hospital stay (*P*<0.001), and in-hospital mortality (*P*=0.035). On the contrary, the RIFLE classification might not be associated with mortality in AKI patients in the ICU. For example, a study in Republic of Korea investigated 256 geriatric patients (≥65 yr old) who developed AKI in the ICU according to the RIFLE classification (17). In this study, the overall in-hospital mortality was 39.8 %, and there were no significant differences in the RIFLE category between survivors and non-survivors. AKIN versus RIFLE classification systems were compared using the SAPS 3 database with regard to outcome (18). Despite presumed increased sensitivity by the AKIN classification, RIFLE shows better robustness and a higher detection rate of AKI during the first 48 h of ICU admission.

We found that AKI developed in patients older than 60 yr of age, length of hospital stay over 6 months, systolic blood pressure less than 100 mm Hg, requirement of mechanical ventilation, relevant comorbidities, anemia, thrombocytopenia, increased serum bilirubin and liver enzymes, and serum sodium abnormalities. Independent risk factors for mortality of AKI included RIFLE class, sepsis, and need for renal replacement therapy, whereas a postsurgical cause of AKI, exposure to nephrotoxins, higher serum creatinine, and urine output were associated with lower mortality risk (9). The risk factors for death among critically ill patients with AKI were hypotension, sepsis, nephrotoxic drug use, respiratory insufficiency, liver failure, hypovolemia, septic shock, multiple organ dysfunction, need for vasoactive drugs, need for mechanical ventilation, oliguria, hypoalbuminemia, metabolic acidosis and anemia (19). In our study, gender, serum potassium, and prescribed drugs were not statistically significant associated with the incidence of AKI. In contrast, age, first serum potassium level, and APACHE II score at admission time is powerful independent predictors of developing AKI in ICU patients (20).

The present study has several limitations that should be considered. First, we specifically focused on patients admitted to ICU. Second, this study was a single-center with a limited sample size and these results may not be generalizable to other ICUs. Third, as a single-center study, there may be bias due to case mix, quality of ICU care, ICU policy, and admission criteria. Fourth, this study represents a snapshot in time and thus this may have led to sampling bias. Finally, we did not attempt to compare RIFLE with other classification systems. However, data from our study have notable similarities and differences in the incidence, risk factor, and outcome of AKI in comparison to other different parts of the world.

## Conclusion

The RIFLE classification is a useful and suitable clinical tool to evaluate the incidence and mortality rate of AKI. In ICU patients, AKI is associated with increased mortality rate. Further prospective and multi-center studies are needed to evaluate the incidence and outcomes of AKI in ICU patients.

## Ethical considerations

Ethical issues (Including plagiarism, informed consent, misconduct, data fabrication and/or falsification, double publication and/or submission, redundancy, etc.) have been completely observed by the authors.
